# The risk of basal and squamous cell carcinomas of the skin cancer incidence and external radiation in the updated National Registry for radiation workers cohort in the UK

**DOI:** 10.1002/ijc.70096

**Published:** 2025-09-01

**Authors:** Nezahat Hunter, Richard Haylock

**Affiliations:** ^1^ Radiation Effects Department UK Health Security Agency (UKHSA), Harwell Campus Oxfordshire UK

**Keywords:** basal cell carcinoma, cohort study, non‐melanoma skin cancer, occupational radiation exposure, squamous cell carcinoma

## Abstract

This study presents the most comprehensive evaluation to date of non‐melanoma skin cancer (NMSC) risk in the UK National Registry for Radiation Workers, examining separately both basal cell carcinoma (BCC) and squamous cell carcinoma (SCC). The cohort includes 172,452 workers with over 5.3 million person‐years of follow‐up (1955–2011) and a mean cumulative dose of 24.9 mSv. Cumulative external radiation was assessed using the personal dose equivalent Hp(10), and Poisson regression estimates the excess relative risk per Sievert (ERR/Sv) of skin cancer incidence. A total of 5748 NMSC cases were identified (4288 BCC, 818 SCC). BCC showed good evidence of a linear dose–response (ERR/Sv = 0.85, 95% CI: 0.39–1.39; *p* <.001), but this weakened after adjusting for anatomical location as a proxy for ultraviolet radiation (UVR) exposure (ERR/Sv = 0.15; 95% CI: −0.17–0.54; *p* = 0.28). However, BCC risk remained elevated among workers who were monitored for internal exposure and those workers first employed before 1960, both of whom experienced higher doses and longer follow‐up. SCC showed only limited evidence of a radiation dose–response (ERR/Sv = 0.87; 95% CI: 0.03–2.05; *p* = 0.04), driven largely by a small number of cases at high dose, and lost significance after adjustment for anatomical location. Most BCC and SCC tumors occurred on sun‐exposed areas, with notably higher BCC risks on the upper limbs. These findings highlight the importance of accounting for UVR exposure in occupational radiation studies of skin cancer and support further research with individual UVR exposure data and skin dose data to better quantify these risks.

## Graphical abstract

What's new?

While ultraviolet radiation from the sun is the main risk factor for non‐melanoma skin cancer (NMSC), additional factors contribute to NMSC development. The present study examined the impact of occupational radiation exposure on NMSC risk among radiation workers in the United Kingdom. For NMSC subtypes, basal cell carcinoma (BCC) risk was elevated in workers who were monitored for internal exposure and for those employed before 1960. Radiation dose‐response was less evident for squamous cell carcinoma. For both subtypes, sun‐exposed areas of the body were most tumour‐prone. Overall, the findings indicate that BCC is most consistently linked to occupational radiation exposure.

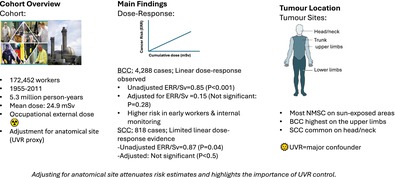

**Abbreviations**
AWE: atomic weapons establishment
BCC: basal cell carcinoma
BEG: British energy generation
BNFL: British nuclear fuels
CCSS: childhood cancer survivor study
CI: confidence interval
ERR: excess relative risk
GE: general electric
Gy: gray
ICD: International Classification of Diseases
ME: Magnox Electric
MoD: Ministry of Defence (MoD),
mSv: millisievert
NHS: National Health Service
NHSCR: National Health Service Central Registers
NMSC: non‐melanoma skinc
NRRW: National Registry for Radiation Workers
RR: relative risks
SCC: squamous cell carcinoma
Sv: sieverts
UKAEA: United Kingdom Atomic Energy Authority
UVR: ultraviolet radiation

## 1 INTRODUCTION

Non‐melanoma skin cancer (NMSC), comprising basal‐cell carcinoma (BCC) and squamous‐cell carcinoma (SCC), is one of the most common types of skin cancer globally, with increasing incidence especially in white Caucasian populations since the 1960s.^1^, ^2^ Ultraviolet radiation (UVR) from the sun is the main environmental risk factor. Intermittent UVR exposure, such as recreational exposure and sunburns, especially during childhood/adolescence, has been linked to BCC, whereas cumulative, chronic exposure in adulthood, including occupational sun exposure, is more strongly associated with SCC.^3^ While these cancers most commonly develop on sun‐exposed areas of the body, BCCs more frequently arise on less exposed areas than SCCs.^2^ Additional risk factors for NMSC include the use of sunbeds, increasing age, genetic susceptibility, immunosuppression, and exposure to arsenic.^3^ The rates for NMSC are highest among fair‐skinned populations, but people with dark skin can still get BCC and SCC at lower rates, and there is often a poorer prognosis among this latter group.^4^

Ionising radiation is an established risk factor for NMSC at high doses, yet its effects at low doses (below 1 Gy) remain unclear. There is also a limited amount of information on the interaction between UVR and ionising radiation in NMSC risk that is not well understood.^5^, ^6^, ^7^, ^8^, ^9^, ^10^, ^11^, ^12^, ^13^, ^14^, ^15^, ^16^, ^17^ While BCC has been more consistently linked with ionising radiation, evidence for SCC is weaker.^3^ The UK National Registry for Radiation Workers (NRRW) is a long‐term occupational cohort ideal for investigating the effects of protracted low‐dose external radiation exposure.^18^, ^19^, ^20^, ^21^, ^22^, ^23^ This study evaluates the associations between occupational external radiation exposures and NMSC subtypes‐BCC and SCC‐in the updated NRRW‐3 cohort, with extended follow‐up of 10 additional years and new analyses incorporating tumour anatomical site data to investigate potential interactions with UVR exposure.

## 2 MATERIALS AND METHODS

### 2.1 The study cohort

Detailed information on the NRRW‐3 cohort design, the study population, data collection, worker characteristics, and follow‐up procedures have been published previously.^18^, ^19^, ^20^, ^21^, ^22^, ^23^, ^24^ Briefly, it is a long‐term cohort study of UK‐based workers employed by major nuclear industry organisations, including British Nuclear Fuels Ltd. (BNFL), the United Kingdom Atomic Energy Authority (UKAEA), the Atomic Weapons Establishment, British Energy Generation of Magnox Electric sites in England and Wales, and Scotland, the Ministry of Defence (MoD), GE Healthcare (formerly Amersham International), and other smaller research/industrial sectors throughout the United Kingdom. The cohort includes both prospective data on workers employed from 1976 onwards—when the study commenced—and retrospective data, covering workers employed as early as the mid‐1940s through to 2011.

Information on vital status, emigrations, and cancer incidence was obtained from the National Health Service Central Registers (NHSCRs), maintained by the National Health Service (NHS) England for England & Wales, and by the National Records of Scotland for Scotland. Mortality data have been collected from 1955, while cancer incidence data have been available nationally since 1971.^25^ NMSC cases were coded using the International Classification of Diseases: ICD‐9 code 173, or ICD‐10 code C44. Tumour histology was coded using the International Classification of Diseases for Oncology (ICD‐O‐3) and further classified by anatomical site; ICD‐10 codes: lip (C44.0); face: eyelid (C44.1); and other unspecified face (C44.3); ear (C44.2); scalp and neck (C44.4); trunk (C44.5); upper limb (C44.6); lower limb (C44.7); and overlapping lesion or not‐specified‐sites (NOS) (C44.8‐C44.9).^26^, ^27^

### 2.2 Workers radiation exposure

This analysis uses external doses that are approximate estimates of Hp(10) doses, which are the dose equivalent in soft tissue at a depth of 10 mm; it is used to estimate how much penetrating radiation is absorbed, mainly from photons such as gamma rays and x‐rays.^18^, ^19^, ^20^ Hp(10) approximates the whole‐body dose (or effective dose), but it tends to be a conservative estimate (an overestimate) because of the body shielding of deeper tissues; however, it is generally adequate for radiological protection. The dose data were recorded using individual dosimeters and reported in sieverts (Sv) or millisieverts (mSv). Hp(0.07) estimates shallow skin dose from beta radiation or low‐energy photons, which is relevant for the skin exposure. However, such skin doses were not routinely collected in the NRRW because employers provided limited information. For the photons of an energy likely to be encountered in the cohort, the Hp(10) dose is a good approximation to the Hp(0.07) dose. Potential exposures to neutrons, beta particles, and tritium were also not considered because of incomplete and uncertain data provided by employers.^22^, ^23^

Approximately 25% of workers were monitored for internal emitters, indicating potential exposure through inhalation or ingestion of radionuclides such as uranium, plutonium, and tritium. Although quantitative internal doses were unavailable, a binary indicator was used to identify workers ever monitored for internal emitters. This monitoring could also involve skin contamination from beta‐emitters. However, the NRRW data lack detailed records to quantify its contribution to Hp(0.07) dose.

The average cumulative dose among workers was 25.5 mSv. Most workers were exposed to relatively low levels of radiation; 62% received cumulative doses under 10 mSv, while 6.2% had moderate to high cumulative doses of 100 mSv or more. Fewer than 1% received doses above 400 mSv, with 70 workers accumulating doses exceeding 1 Sv over their working lifetimes.^17^, ^18^, ^19^, ^20^, ^21^, ^22^, ^23^ Among those with doses above 400 mSv, 84% were also monitored for internal emitters and had longer employment durations (25+ years) and started work during earlier periods when annual occupational external doses were generally higher. The mean external dose among internally monitored workers was 61 mSv, compared with 13 mSv for those not monitored for internal exposure. However, dose estimates from earlier study periods, especially for internally monitored workers, carry greater uncertainty.^23^

### 2.3 Statistical methods

Poisson regression models were used to estimate excess relative risk (ERR) per sievert (Sv) to investigate the association between the incidence of BCC and SCC and cumulative external radiation dose among UK nuclear workers. The linear ERR dose–response model is expressed as:

$$\lambda \;\left(a,b,g,f,s,l,d\right)=\lambda_{0}\;\left(a,b,g,f,s,l\right)\;\left[1+\mathrm{ERR}\;\left(d\right)\;\varepsilon \left(.\right)\;\right]$$

where *λ*₀ is the baseline rate depending on attained age (*a*), birth cohort (*b*), sex (*g*), facility (*f*), employment category (*s*), and anatomical site (*l*), and *d* represents cumulative dose. ERR(*d*) describes the dose–response function of cumulative dose, and *ε*(.) models effect modification.

A linear dose–response (ERR(*d*) = *βd*) was the main model. A linear‐quadratic model (ERR(*d*) = *β*₁*d* + *β*₂*d* ^2^) was also used to assess curvature in the dose–response relationship. Effect modifiers considered included sex, attained age, and age at first exposure (defined as the age when a worker received their first monitored dose). Data were structured into person‐years tables stratified by relevant variables, with each cell containing the number of person‐years and incident cases.

Baseline hazard functions λ_0_ were examined using fully parametric, semiparametric, and stratified approaches, with the fully parametric model presented here due to similar findings across models.^23^ Follow‐up started from the latest of employment start, availability of full dose data, or 1 January 1971, and ended at cancer diagnosis, death, 85th birthday, or 31 December 2011. In total, the cohort contributed over 5.25 million person‐years of observation. For each worker, person‐years‐at‐risk were accumulated over time from the start of follow‐up, defined as the latest of the date of start of radiation work with a participating employer, the date from which full dose data were available, or 1 January 1971 until the earliest of the date of diagnosis of the first primary cancer, the date of death, their 85th birthday, or 31 December 2011. In total, the cohort contributed over 5.25 million person‐years of observation. A 10‐year exposure lag was used to account for latency and facilitate comparison with earlier NRRW studies.^18^, ^19^, ^20^ Sensitivity analyses tested alternate lag periods of 5, 15, and 20 years.

Since individual‐level (UVR) data were unavailable in the NRRW, the anatomical location of skin cancer was used as a proxy measure for UVR exposure in the analysis. Tumour locations were categorised by likely UVR exposure: high (face: including eyelid and other face areas, scalp/neck, ear); high‐to‐medium (lip, upper limbs); medium (lower limbs); low (trunk); and unspecified/overlapping. This stratification was included in the baseline model to adjust for UVR exposure. These nine categories were included in the baseline model through stratification to adjust for UVR exposure.

Subgroup analyses were conducted separately for workers who only experienced external exposure and those who were additionally monitored for internal exposure to explore whether internal monitoring status modified the dose–response relationship.

All statistical analyses were conducted using the AMFIT module in the EPICURE software.^28^ Confidence intervals (CIs) and likelihood ratio tests were used to evaluate statistical significance, with two‐sided P‐values and a 5% significance.

## 3 RESULTS

The dataset used in this study is similar to that used in previous reports investigating other health outcomes in relation to external radiation doses,^19^, ^20^, ^21^, ^22^ comprises 172,452 workers (90% men), with a total of 5.3 million person‐years of follow‐up from 1955 to the end of 2011. Table 1 summarizes cohort characteristics by first employer and cumulative dose. The average follow‐up was 30 years, with the longest durations among workers from UKAEA, BNFL, and British Energy Generation & Magnox Electric (BEG & ME).

**TABLE 1:** NRRW‐3.5 cohort.

	MoD	BNFL	UKAEA	BEG and ME	AWE	GE Healthcare	Rolls‐Royce	Research Organ	Total
Follow‐up period	1961–2011	1948–2011	1946–2011	1959–2011	1948–2011	1976–2011	1959–2011	1957–2011	1946–2011
Cohort size (male %)	63,627 (89.5)	40,071 (91.9)	27,502 (90.9)	16,308 (97.6)	14,730 (88.6)	3871 (60.2)	3265 (91.0)	3078 (89.4)	172,452 (90.3)
Person‐years follow‐up (unlagged)	1,810,272	1,256,556	954,727	517,277	426,408	101,472	92,306	93,901	5,252,919
Mean length of follow‐up (years)	28.5	31.4	34.7	31.7	28.9	26.2	28.3	30.5	30.5
Mean period of birth	1942	1935	1933	1939	1933	1944	1945	1938	1936
Mean period of starting work	1975	1967	1966	1973	1967	1977	1976	1970	1975
Mean external dose in mSv (max)	8.0 (1184)	54.7 (1875)	34.3 (1677)	24.5 (1219)	8.2 (629)	32.5 (1591)	14.7 (1547)	10.9 (538)	24.9 (1875)
No. of NMSC cases (unlagged)	1680	1461	1150	643	487	102	98	127	5748
No. of BCC cases	1344	1157	866	514	372	83	75	104	4515
No. of SCC cases	222	213	201	90	68	13	15	17	839
No. of other NMSC cases	114	90	83	39	47	6	8	7	394

The highest average radiation doses were observed among BNFL, UKAEA, and GE Healthcare, reflecting earlier employment periods with higher typical exposures. About 60% of the cohort was born before 1950, and 61% began work before age 30 years (mean starting age: 30 for men and 27 for women). Overall, 63% of the cohort had a follow‐up exceeding 25 years, and 38% were followed to at least age 65. By 31 December 2011, a total of 5 748 cases with NMSC had been registered; of these, approximately 78% BCC, 15% SCC, and 7% other types of NMSC, all registered as the first primary cancer.

### 3.1 Basal cell carcinoma (BCC)

A total of 4,288 BBC cases were registered as a first primary cancer at least 10 years after follow‐up began. Mean age at diagnosis was 65, with 71% diagnosed after the age of 60. Most BCC cases (65%) occurred between 2000 and 2011, and only 5% before 1985. Among these cases, 90% of workers had cumulative doses <100 mSv, while 1.8% exceeded 400 mSv.

**FIGURE 1:**
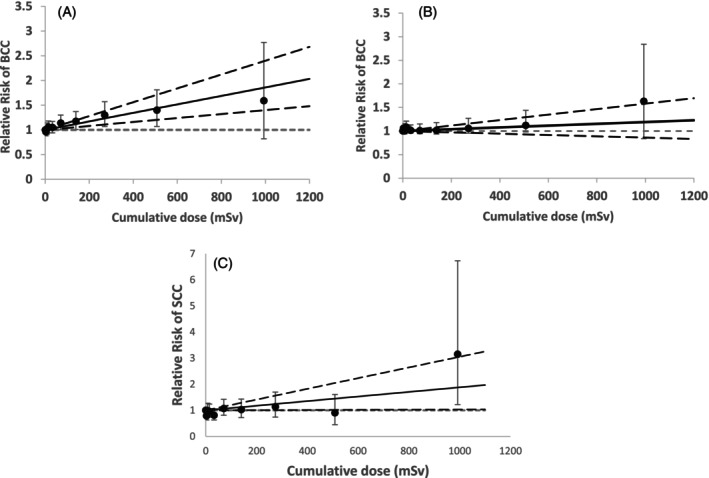
Relative risk (RR = 1 + ERR) of BCC and SCC in relation to external radiation with 10 dose‐category‐specific estimates and the linear trend (and 95% CI) based on 10‐year‐lagged period for dose–response; (A): No adjustment for BCC anatomical location, (B): Additional adjustment for BCC anatomical location in the baseline model, (C): No adjustment for SCC anatomical location.

A statistically significant linear dose–response relationship (Figure 1A) was found between cumulative external radiation dose and BCC risk (*p* < 0.001; ERR/Sv = 0.85, 95% CI; 0.39–1.39). This significance remained when the data was restricted to cumulative doses <100 mSv (*p* = 0.02, ERR/Sv = 2.10, 95% CI: 0.31–4.07), but weakened at the dose range < 50 mSv(*p* = 0.06, ERR/Sv = 3.07, 95% CI: −0.13, 6.59). However, the association in the full dataset became non‐significant (ERR/Sv = 0.15, 95% CI: −0.17; 0.54) (Figure 1B) after adjusting for anatomical site (proxy for UV exposure), suggesting potential confounding by ultraviolet radiation (UVR). No evidence of nonlinearity based on the linear‐quadratic (LQ) model was observed (*p* >.5). Alternative lag periods of 5, 15, or 20 years had little impact on the results (Table 2) with or without adjustment for anatomical site and were similar to those using a 10‐year lag.

**TABLE 2:** The interaction effect between radiation and various temporal factors of interest for BCC and SCC (lagged by 10 years), having adjusted baseline factors (no adjustment for anatomical locations in the baseline model).

	BCC			SCC	
	Cases	ERR/Sv (95% CI)^a^	ERR/Sv (95% CI)^b^	Cases	ERR/Sv (95% CI)^a^
Overall	4288	0.85 (0.39; 1.39)	0.15 (−0.17; 0.54)	818	0.87 (0.03; 2.05)
Sex					
Male	4031	0.84 (0.38, 1.37)	0.16 (−0.11; 0.50)	789	0.89 (0.03; 2.08)
Female	257	5.79 (−1.33; 15.7)	−0.88 (−0.82; 1.34)	29	−0.79 (−0.84; 22.1)
*p*‐value^c^		0.17	>0.5		0.25
Attained age (years)					
<55	776	0.68 (−0.53; 2.47)	0.22 (−0.61; 1.46)	53	0.69 (−5.39; 10.1)
55–59	1144	0.56 (−0.26; 1.56)	−0.28 (−0.78; 0.31)	154	−0.56 (−0.60; 2.88)
65+	1516	0.87 (0.25; 1.62)	0.19 (−0.19; 0.64)	323	0.80 (0.06; 2.57)
75+	852	1.10 (0.30; 2.08)	0.55 (−0.09; 1.35)	288	1.07 (0.46; 2.72)
*p*‐value^c^		>0.50	0.46		>0.50
Age‐at‐first exposure (years)					
<25	1011	1.66 (0.73; 2.78)	0.09 (−0.36; 0.69)	15	12.7 (−2.56; 37.8)
25–29	903	−0.09 (−0.56; 0.57)	0.19 (−0.39; 0.71)	279	−0.16 (−0.89; 1.00)
30–39	1339	1.37 (0.59; 2.28)	0.29 (−0.24; 0.71)	283	2.01 (0.49; 4.11)
40+	1035	0.88 (−0.17; 2.22)	0.28 (−0.40; 1.29)	241	1.95 (−0.16; 5.19)
*p*‐value^c^		0.003	>0.50		0.02
Time since first exposure (years)					
10–20	679	−0.60 (−1.00; 1.72)	−0.59 (<−0.65; >0.59)	87	9.0 (1.74; 21.0)
20–29	1271	1.65 (1.09; 3.02)	0.09 (<−0.13; >0.78)	182	2.24 (−0.98; 675)
30–39	1319	0.52 (0.25; 1.23)	0.33 (<0.29; >0.36)	281	0.96 (−0.26; 2.86)
40+	1019	1.00 (0.72; 1.31)	0.05 (<−0.06; 0.45)	268	0.45 (−0.42; 1.77)
*p*‐value^c^		0.15	0.36		0.10
Start of employement (calendar year)					
<1960	975	0.90 (0.36; 1.55)	0.50 (0.04; 1.06)	239	0.53 (−0.27; 1.78)
1960–1969	1309	0.75 (−0.06; 1.74)	0.04 (−0.54; 0.57)	274	0.23 (−1.36; 2.40)
1970–1979	1300	0.36 (−0.56; 1.54)	−0.63 (−0.63; −0.00)	220	3.48 (0.41; 7.99)
1980+	704	8.18 (3.62; 13.9)	−0.37 (−0.64; 1.17)	85	8.79 (−0.70; 25.1)
*p*‐value^c^		0.004	0.05		0.08
Lagging periods					
Lag 5		0.81 (0.37; 1.32)	0.20 (−0.12; 0.58)		0.84 (0.01; 1.98)
Lag 15		0.93 (0.43; 1.50)	0.17 (−0.18; 0.58)		0.89 (0.01; 2.10)
Lag 20		1.01 (0.46; 1.64)	0.14 (−0.23; 0.59)		0.84 (−0.09; 2.14)

^a^ Baseline model adjusted for sex, attained age, birth year, first employment and industrial classification.

^b^ Baseline model adjusted for sex, attained age, birth year, first employment and anatomical locations.

^c^ Test of heterogeneity of the ERR/Sv across categories.

Effect modification analysis (Table 2) showed no significant variation in BCC risk by sex or time since first exposure. However, there was strong evidence that age at first radiation exposure modified the dose–response relationship (*p* = 0.003); although no clear dose–response pattern emerged, workers first exposed before age 25 had the highest risk (ERR/Sv = 1.66; 95% CI: 0.73–2.78), followed by those first exposed at ages 30–39 (ERR/Sv = 1.37; 95% CI: 0.59–2.28). Calendar period of first employment was also a significant modifier of BCC (*p* = 0.004); among workers first hired after 1980 had the greatest risk (ERR/Sv = 8.18; 95% CI: 3.62–13.9), while those employed before 1960 had a smaller but still statistically significant excess risk (ERR/Sv = 0.90; 95%CI: 0.36–1.55). However, both age at first exposure and year of first employment effects lost significance after anatomical site adjustment.

**FIGURE 2:**
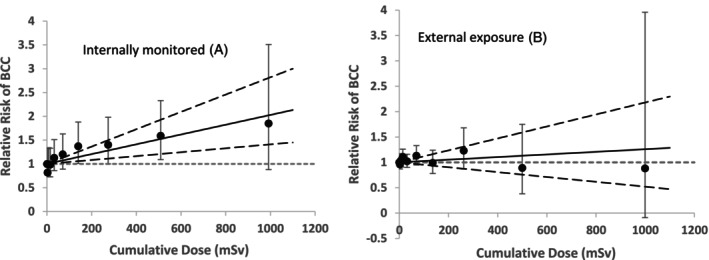
Relative risk of BCC in relation to external radiation with 10 dose‐category‐specific estimates and the linear trend estimate with 95% CI by 10‐year‐lagged period for the sub‐cohort of workers: (A): Also potentially monitored for internal exposure; (B): External radiation workers sub‐cohort.

For the sub‐cohort of workers monitored for internal exposure, clear evidence for a linear dose–response trend was found (*p* = 0.012; ERR/Sv = 1.03; 95% CI: 0.41–1.82) (Figure 2A), which was not statistically significant when the data were restricted to <100 mSv (*p* = 0.15). The trend persisted in the full dataset even after adjustment for anatomical site for this sub‐group, although the risk estimate was reduced by half (*p* = 0.03, ERR/Sv = 0.52; 95% CI: 0.03–1.14). This finding was based on a small number of cases (*n* = 10 cases) who had higher cumulative dose (above 800 mSv) and longer employment duration (more than 30 years). The BCC cases in this group were located at the lip (2 cases), external ear (5 cases), trunk (1 case), and overlapping sites (2 cases). When restricting the analysis to workers with doses <800 mSv, the trend was no longer statistically significant (*p* = 0.10, ERR/Sv = 0.46; 95% CI: −0.08, 1.14). As a result, the significant dose–response observed in the main analysis of BCC appeared to be influenced by those workers monitored for internal exposure, although only 27% of all BCC cases occurred in that group (including 48 female cases). For the external radiation workers sub‐cohort (Figure 2B), with the same adjustment, the risk estimate became negative (ERR/Sv = −0.59, 95% CI: −0.61; 0.01, *N* = 3130 cases), but the evidence was weak (*p* = 0.06). In no instance did the LQ model fit better than the linear model (*p* > 0.5).

### 3.2 Squamous cell carcinoma (SCC)

There were 818 SCC cases (mean age 70; 87% diagnosed >60 years). Most (70%) were diagnosed after 2000; only 4% of cases were diagnosed before 1985. About 89% had doses <100 mSv; only 2.1% exceeded 400 mSv. A total of 28% were monitored for internal exposure, including only 2 female cases.

Evidence for a linear trend was found (*p* = 0.04; ERR/Sv = 0.87; 95% CI: 0.03–2.05) (Figure 1C), but this result was driven by six high‐dose cases (>800 mSv), five of whom were monitored for internal exposure and had longer employment durations of more than 25 years. Of these six cases, two were located on the face, one on the ear, one on the trunk, and two overlapping areas. The linear‐quadratic (LQ) model did not improve model fit (*p* > 0.50). When the dose range was restricted to 0–800 mSv, the evidence for a dose–response trend was lost (*p* = 0.42). After adjusting for anatomical location over the full dose range, there was no evidence for a linear trend (*p* = 0.32; ERR/Sv = 0.37; 95% CI: −0.27 to 1.30), nor of an LQ trend (*p* > 0.5).

There was some evidence of effect modification by age‐at‐first exposure and calendar period (Table 2); a significant increase in risk was observed in those first exposed between ages 30–39 and the greatest effect among workers first employed after 1980. Modest evidence for a trend was also found in the period 10–20 years after first exposure, but no significant trend was seen in later time periods. However, when anatomical site was included in the baseline model, there was no evidence of effect modification for any of these factors (not shown here). No consistent trends were observed across lag periods (Table 2).

When analyzed separately by sub‐cohort, no evidence of radiation‐associated excess risk for SCC was found in either external exposure experienced only workers (*p* > 0.5; ERR/Sv = 0.006; 95% CI: −0.10 to >0.11) or those monitored for internal exposure (*p* = 0.15; ERR/Sv = 0.76; 95% CI: −0.20 to 2.43). Adjusting for the anatomical location of SCC had no effect on the results in either sub‐cohort.

### 3.3 Anatomical Site and radiation

BCC was most common on UV‐exposed areas (Table 3), particularly on the ear (38.7%), the scalp/neck (12%), eyelid (6.2%), and on other face areas (6.6%). Less frequent sites included lip (6.0%), upper limbs (3.1%), lower limbs (1.3%), and trunk (5%), with 21.1% classified as overlapping or unspecified. Crude BCC incidence rates increased with cumulative radiation dose in sun‐exposed areas (lip, face, ear and scalp/neck), and the highest trunk rate was seen in workers exposed to over 400 mSv (see Supporting Information Table S1).

**TABLE 3:** Excess relative risk (ERR/Sv) of BCC of the skin across categories of anatomical sites based on linear model in relation to external doses lagged 10 years.

Sites (ICD‐10 codes)	BCC (full data)			BCC (internal workers sub‐cohort)		
	Cases (female)	ERR/Sv (95% CI)^a^	ERR/Sv (95% CI)^b^	Cases (female)	ERR/Sv (95% CI)^a^	ERR/Sv (95% CI)^b^
Lip (C44.0)	257 (21)	0.34 (−0.77; 2.45)	0.85 (−0.44; 2.80)	75 (6)	0.68 (−0.77; 4.46)	1.08 (−0.53; 4.16)
Face: eyelid (C44.1) and other face (C44.3)	549 (27)	0.05 (−0.72; 1.19)	0.12 (−0.63; 1.12)	160 (7)	0.36 (−0.76; 2.55)	0.65 (−0.43; 2.41)
Ear (C44.2)	1658 (105)	−0.22 (−0.53; 0.24)	−0.19 (−0.51; 0.26)	481 (18)	−0.04 (−0.47; 0.63)	0.02 (−0.43; 0.67)
Scalp and neck (C44.4)	509 (20)	1.09 (−0.39; 3.33)	0.64 (−0.55; 2.33)	130 (3)	1.16 (−0.07; 5.01)	1.02 (−0.55; 3.82)
Trunk (C44.5)	25 (8)	0.29 (−0.80; 2.45)	0.40 (−0.68; 2.17)	48 (3)	0.78 (−0.84; 6.06)	0.76 (−0.70; 3.83)
Upper limbs (C44.6)	135 (17)	6.60 (0.97; 17.6)^d^	7.07 (2.08; 14.8)	38 (0)	26.8 (2.38; 206)^d^	6.46 (0.48; 22.5)
Lower limbs (C44.7)	54 (0)	0.46 (−2.03; 18.2)	0.58 (−1.78; 5.98)	14 (0)	16.4 (<−28.2; 446)	1.39 (−1.74; 12.0)
Overlapping lesion (C44.8)	873 (56)	0.47 (−0.32; 1.62)	0.34 (−0.36; 1.34)	201 (11)	1.50 (0.01; 4.10)	1.49 (0.07; 3.68)
NOS (C44.9)	38 (3)	1.44 (−1.35; 16.2)	5.51 (0.11; 17.4)	11 (0)	1.44 (−3.34; 40.3)	7.74 (−2.55; 47.0)
Test for heterogeneity^c^		‐	*p* = .024		‐	*p* = .19
Total	4288 (257)	0.85 (0.39; 1.39)		1158 (48)	1.03 (0.41; 1.82)	

^a^ Risk estimates based on restricted to only those workers with specific anatomical sites.

^b^ Adjusted for anatomical site over the full data.

^c^ Test of heterogeneity of the ERR/Sv across categories (significant at *p*<.05).

^d^ *p* = .012.

SCC also appeared mostly on sun‐exposed sites: ear (23.1%), eyelid (21.4%), other face areas (14.3%), and lip (2.8%). Other sites included the trunk (13.6%), scalp/neck (8.3%), upper limbs (5.8%), and lower limbs (0.8%), with 9.9% unspecified. SCC rates increased with radiation dose for the face, ear, scalp/neck, and were notably higher on the trunk at doses above 10 mSv (see Supporting Information Table S2). Anatomical site distributions by dose level are shown in Supporting Information Figure S1.

When the analyses were restricted to specific anatomical sites (Table 3), the strongest and only statistically significant ERR/Sv was seen for BCC on the upper limbs (*p* = 0.012). Positive, albeit non‐significant, risk estimates were observed for the lip (*p* > 0.5), scalp/neck (*p* = 0.18), trunk (*p* > 0.5), and lower limbs (*p* > 0.5), while risks for the face (including eyelid and other face) were small (*p* > 0.31) and the ear was negative (*p* = 0.31). A test of heterogeneity indicated good evidence for variation in BCC risk across the nine anatomical sites (*p* = 0.02), with the upper limbs showing a notably stronger association with radiation than other body regions. The risk estimates were positive but not statistically significant for the lip, scalp/neck, lower limbs, and trunk, suggesting a possible link with radiation dose. In contrast, risk estimates for the face and the ear showed little or no increase in risk, indicating limited effects of radiation in those areas.

Across a restricted dose range of 0–500 mSv, good evidence was observed for an excess of BCC on the upper limbs only (ERR/Sv = 6.24, 95% CI:1.39, 13.83), with positive risk estimates for the lip (ERR/Sv = 0.84, 95% CI: −0.81, 3.23) and lower limbs (ERR/Sv = 0.55, 95% CI: −1.79, 5.90) and a negative risk estimate for the face (ERR/Sv = −0.09), ear (ERR/Sv = −0.64), and trunk (ERR/Sv = −0.16) and zero risk for scalp/neck (ERR/Sv = 0.005); although in contrast to the unrestricted analyses, there was no evidence for heterogeneity across anatomical sites (*p* = 0.11). Further restriction to lower dose ranges (0–100 mSv) removed all evidence of heterogeneity (*p* > 0.5); although positive, albeit non‐significant, excess risk estimates were only observed for lower (ERR/Sv = 5.39) and upper limbs (ERR/Sv = 5.10) and lip (ERR/Sv = 0.12).

Among internally monitored workers, the site‐specific risk pattern for BCC was like that of the full cohort. The highest ERR/Sv again occurred in the upper limbs, while other sites showed non‐significant positive associations. The ear continued to show little or no increase in risk. Heterogeneity in BCC risk by anatomical site among this subgroup was not statistically significant (*p* = 0.19), suggesting a relatively uniform distribution of radiation effects after accounting for anatomical site. No interaction between radiation dose and body site was found in the sub‐cohort exposed only to external radiation (see Supporting Information Table S3).

In contrast, no statistically significant heterogeneity between anatomical sites was observed for SCC, although the higher positive estimates were found only for scalp/neck, and negative estimates for the lip, upper and lower limbs (Supporting Information Table S3).

## 4 DISCUSSION

This is the first study to assess NMSC risk in the NRRW‐3 cohort by tumour subtype (BCC and SCC) and anatomical site in relation to cumulative external radiation exposure. Compared to the earlier NRRW‐3 study,^18^, ^19^ follow‐up was extended by 10 years, increasing NMSC case numbers from 326 to 5 480 and substantially improving statistical power. This substantial increase likely suggests enhanced case ascertainment due to occupational health surveillance, where more frequent or thorough medical monitoring can lead to higher detection rates. However, NMSC registration completeness is often incomplete, which could distort the dose–response relationship if case capture varies by geographical location and cumulative dose. This is especially relevant for retired workers who are no longer covered by occupational medical care.

The previous studies reported evidence for an increasing linear trend for all NMSC combined; the ERR/Sv for the original NRRW‐3 study^19^ was 1.50 (95% CI: 0.05–3.85, *n* = 326) and for the updated NRRW‐3,^20^ it was 0.88, (95% CI: 0.47–1.34, *n* = 5460), consistent with our subtype specific findings.

A key strength of this study is the large number of BCC and SCC cases and the availability of anatomical site information, allowing adjustment for UVR exposure using anatomical site as a proxy, which is a common approach when individual UVR data are unavailable, as is the case in the NRRW. Incorporating anatomical site in models helped account for potential UV‐related confounding, strengthening the interpretation of radiation‐related risk estimates.

Adjusting for anatomical site weakened the radiation–skin cancer association in the full cohort and also in some subgroups, indicating potential UVR confounding. Elevated risks were initially observed among workers first hired after 1980 (who had lower doses and shorter follow‐up); but this result reversed after site adjustment, indicating residual confounding by UVR exposure. In contrast, workers hired before 1960, with higher doses and longer follow‐up, retained a raised BCC risk after adjustment, showing a stronger occupational radiation effect in earlier cohorts. Elevated BCC risk persisted after adjustment for anatomical site among the internally monitored workers, who comprised 27% of all BCC cases and likely had higher external doses and longer employment. However, no clear dose–response was seen among externally exposed workers who had generally lower doses and shorter careers. This contrasts with a recent NRRW solid cancer study,^23^ which found a positive trend in external workers and a non‐significant negative trend in internal workers, possibly reflecting cancer type or exposure differences.

Higher BCC risk among internally monitored workers may reflect unaccounted for exposure to low‐energy beta radiation, which affects skin but is poorly represented by Hp(10), which measures deep tissue dose. Although Hp(10) may underestimate skin dose for low‐energy beta radiation from shallow‐penetrating radiation, it is considered a good proxy in photon‐dominated exposures, but may not be so for some internally monitored workers in the NRRW. Work conditions, exposure geometry, and use of protective equipment and work activities can affect actual skin dose,^29^ but the use of Hp(10) likely does not introduce major bias. Still, the risk estimates align with other studies using direct skin dose, suggesting Hp(10) provides broadly comparable results.

Previous studies also support the link between BCC risk and ionising radiation. The results from this study were compared to others that generally used direct skin dose in Table 4. Despite this, the confidence intervals generally overlapped, and all studies reported BCC as the NMSC type most associated with radiation. However, members of the Mayak cohort were generally exposed to higher doses (often >1 Sv), while the NRRW exposure and that of the US study of female radiographers were lower.

**TABLE 4:** Comparison risk estimates of the ERR/Sv at 1 Sv or Gy for external radiation exposure among published studies.

Study period	Cohort size	Mean dose (external radiation)	Basal cell carcinoma (BCC)		Squamous cell carcinoma (SCC)	
			No. of cases	ERR/Sv or Gy (95% CI)	No. of cases	ERR/Sv or Gy (95% CI)
*Present NRRW‐3.5 workers study* (*UK*) (x‐ray, gamma, internal radiation)						
0.85 (0.39; 1.39) a						
1955–2011	172,452 (90% male)	24.9 mSv (Gamma rays)	4288 (94% male)	0.15 (−0.17; 0.54) a1	818 (96% male)	0.87 (0.03; 2.05) a
				1.03 (0.41; 1.82) b		0.37 (−0.27; 1.30) a1
				0.52 (0.03; 1.14) b1 0.26 (−0.48; 1.18) b2		0.41 (−0.50; 1.66) b3
*Mayak PA radiation workers study* (*Russia*) (gamma, internal radiation)						
1948–2018	22,377 (75% male)	500 Gy (skin dose; gamma rays and alpha radiation)	289 (63%male) c	0.59 (0.25; 1.09) c	48 (68% male) c	0.13 (−0.24; 0.90) c
			53 c1	1.30 (0.27, 3.29) c1	12 c1	0.63 (−0.37; 3.25) c1
			83 c2 51 c3 289 c4	0.74 (0.19, 1.97) c2	14 c2	0.52 (−0.63; 10.3) c2
				0.47 (n/a; 2.15) c3 0.50 (0.22; 0.91) c4	7 c3 5 c4	−0.18 (n/a; 1156) c3 −0.07 (−0.73; 1.78) c4
*Atomic Bomb Survivors* (*Japan*) (gamma, neutrons)						
1958–1996	80,158 (40% male)	330 mGy (skin dose; mainly gamma rays)	123 (37% male)	2.0 (0.69; 4.30) d −0.05 (<−0.05, 1.2) d1	114	−0.12 (<−0.12; 0.25)
					64	0.71 (0.06; 1.9) e
1958–1998	105,427		166 (37% male)	2.2 (0.78; 2.90) * d2	‐	‐
				0.48 (0.12; 1.3) * f 2.64 (2.2; 3.0) * f1		
*Medical Workers, Radiologic technologists* (*USA*) (x‐ray radiation)						
1983–2005	65,719 (25% male)	55.8 mGy (skin dose; x‐rays)	3615 (22% male)	0.03 (−0.39; 0.56) g	‐	‐
				0.59 (−0.11; 1.42) h1 2.92 (1.39; 4.45) h2		
*Patients irradiated for tinea capitis* (*Isreal*) (x‐ray radiation)						
1950–1980	10,834 (1–15 years old; 50% male)	6.1 Gy (x‐rays)	54	0.70 (0.35; 1.32)	‐	‐
*Patients irradiated for tinea capitis* (*USA‐New York*) (x‐ray radiation)						
50 years	2224 (1–15 years old; 88% male)	4.3 Gy (x‐rays)	124	0.60 (0.3; 1.1) i	‐	‐
*Childhood cancer survivors* (*USA*) (radiotherapy)						
1994–2003	12,858 (1–20 years old; 54% male)	0.01–63 Gy	199 (53% male)	1.09 (0.49; 2.64)	‐	‐

*Note*: a: The full cohort; a1: The full cohort with additional adjustment for anatomical site; b:Monitored for internal workers sub‐cohort; b1: Monitored for internal workers sub‐cohort with additional adjustment for the BCC site; b2: External workers sub‐cohort; b3: Restricting to dose below 800 mSv; c: The full cohort; c1:The plutonium production plant, workers exposed both external and internal radiation; c2: The radiochemical plant, workers exposed both external and internal radiation; c3: Reactor plant, workers exposed only external radiation; c4: Results for NMSC and based on Hp(10) dose; d: Slope over threshold dose of 0.63 Gy, based on linear‐threshold model with effect modifiers (at age 70 after exposure at age 30 at exposure); d1: below the threshold dose of 0.63 Gy; *: 90% CI; d2: Slope at 1 Gy, based on linear model without effect modifiers; e: SCC in situ (is a surface form of skin cancer); f: Based on spline model for doses less than 1 Gy; f1: Based on spline model doses above 1 Gy; g: Overall estimate using the full cohort; h1: Age at exposure less than 30 years; h2; First exposed before 1960; i: Caucasian.

In the Japanese A‐bomb survivor cohort, BCC risk increased above 1 Gy and persisted for decades following acute radiation exposure.^5^, ^6^ A threshold around 0.63 Gy^5^ was suggested (ERR/Gy = 2.0; 95% CI: 0.69–4.30), implying risks increase sharply at higher doses and may be minimal below this point; these higher risks are likely due to the acute, high‐dose nature of exposure. Similarly, occupationally exposed Russian Mayak workers who received higher doses also showed increased BCC risk,^7^, ^8^ with skin dose estimates raising risk by approximately 18% over Hp(10), indicating the measurement method may not drastically affect results (Table 4). This study found no clear evidence of a linear‐quadratic or threshold effect for BCC or SCC, either in the entire cohort, in sub‐groups, or when analyses were restricted to workers with external doses under 100 mSv (Figure S2 in the supporting information).

A US study of mostly female radiographers^11^, ^12^ showed increased BCC risk only in those exposed before age 30 and before 1960, when radiation protection was less strict. No excess risk was found after 1960, aligning with reduced doses due to improved safety. Overall, their ERR/Gy (0.03) was lower than in other studies, consistent with lower exposure levels.

Radiotherapy studies show strong links between BCC and radiation treatment, especially in patients treated at younger ages with high localised doses and the risk continuing to rise long after exposure.^9^, ^10^, ^30^, ^31^, ^32^, ^33^, ^34^ However, these patients received much higher localised doses than occupationally exposed workers or atomic bomb survivors.

In contrast to BCC, the association between SCC and radiation exposure is less consistent (Table 4). In A‐bomb survivors, only in situ SCC (Bowen's disease) showed dose response. Most radiotherapy studies found no excess SCC risk, apart from a New Hampshire study.^30^, ^31^ In the NRRW, a possible increase in SCC risk was noted at high doses (above 800 mSv), but this was based on very few cases, limiting conclusions.

### 4.1 Potential factors affecting NMSC and ionising radiation

Our findings suggest internally monitored workers contributed significantly to the observed BCC–radiation association. It is more likely that skin contamination by beta‐particle emitters would provide the skin dose rather than doses from intakes of radionuclides. These individuals had higher external doses and longer employment. A potential explanation for higher UVR exposure among this group could be socioeconomic: longer employment and higher salaries might allow more travel to sunny destinations.^35^ However, without individual UVR or travel data, this remains speculative. Given that UVR is a major cause of NMSC, potential confounding by UVR exposure must be considered when interpreting the observed increased risk of BCC in this subgroup.

Internal exposure itself may also contribute to BCC risk, though evidence is limited. The only known study on internal emitters and NMSC examined Czechoslovak uranium miners, reporting a significant excess of BCC on the face and neck linked to alpha particle exposure from radon,^36^, ^37^, ^38^, ^39^ but possible confounding by arsenic exposure and poor medical record quality weakens these findings.^35^ Other uranium miner studies or ecological studies on residential radon exposure and NMSC show inconsistent or weak associations due to methodological limitations.^40^, ^41^, ^42^

Younger age at radiation exposure has been associated with higher NMSC risk in both the A‐bomb and radiotherapy cohorts.^5^, ^9^, ^10^, ^31^ Unlike these acute, high‐dose exposures, NRRW exposure is protracted and cumulative over potentially many years. In our study “age at first monitoring” was defined as a proxy for first exposure, generally marking the start of employment in a radiation‐related work. It was used to investigate whether earlier start of radiation exposure is associated with elevated BCC risk. Higher BCC risk was seen in workers first exposed before age 25 and in those aged 30–39 compared to other age groups, but this disappeared after anatomical site adjustment, again pointing to UVR confounding. In the Mayak cohort, BCC risk was also slightly higher in those first employed after age 30, though not significantly so.^7^, ^8^

NMSC is primarily caused by cumulative UVR exposure, with a lighter skin complexion, individual UV sensitivity, and frequency of sunburn as additional risk factors. Among white Caucasians, BCC is more common than SCC, consistent with the NRRW cohort.^43^, ^44^ Most BCC and SCC cases in the NRRW occurred on sun‐exposed body sites, and higher ERR/Sv estimates for both cancer types were generally seen at these locations. However, as noted earlier, Hp(10) may not reflect true skin doses at sun‐exposed locations like the face or forearms, due to shielding, clothing, and work practices. According to ICRP 116,^29^ skin dose varies by radiation type and angle of exposure, especially for beta radiation. Hence, site‐specific risks may reflect both UVR influence and limitations in skin dose estimation.

Our anatomical site analysis provides insight into the relationship between ionising radiation and BCC risk. Good evidence for a radiation‐associated excess BCC risk was found only for the upper limbs, with positive but non‐significant estimates for the lip, scalp/neck, trunk, and lower limbs. No excess was observed for the face (including the eyelid and other face) and ear. Significant heterogeneity across sites (*p* = .02) supports the hypothesis that local UVR exposure or radiation dose variation affects risk. These patterns were more evident at higher doses above 500 mSv and among internally monitored workers, suggesting a complex interaction between radiation, local UV exposure, and site‐specific exposure. This underlines the need for refined dose assessment in future studies.

Comparison with the A‐bomb survivor cohort reveals methodological and interpretative differences. That study found higher BCC risk in UV‐shielded areas,^5^, ^45^ though the difference was not statistically significant; a markedly higher excess absolute risk (EAR) was seen in UV‐exposed areas based on a small number of cases, suggesting a possible additive radiation effect without clarifying the nature of interaction with UVR.^5^ In contrast, our study, using more detailed anatomical classification, found the highest BCC risks in typically sun‐exposed areas. These discrepancies may reflect differences in background cancer rates, radiation exposure type (acute vs. chronic), use of skin doses, and skin pigmentation by the A‐bomb survivor or anatomical site definitions. Our findings suggest that UV‐related confounding may obscure or modify radiation effects, underscoring the need for precise anatomical classification and sun exposure adjustment in occupational studies of skin cancer.

Radiotherapy studies also highlight how both local radiation dose and UVR exposure influence BCC risk. In the Israeli tinea capitis cohort,^9^ BCC was most common on the scalp, where radiation dose was highest. The New York tinea capitis study^8^ found BCC mainly in light‐skinned children, with none in dark‐skinned children, suggesting skin tone matters. Both studies linked higher BCC risk to fair skin and histories of severe, intermittent sunburn. The Childhood Cancer Survivor Study reported the most BCCs in Hodgkin lymphoma survivors appeared on the back, chest, or neck, which were the most common radiation treatment areas, and was also stronger among those with BCC on the head or face (mainly from survivors of leukaemia and brain tumours) than among those with BCC in other locations.^33^ In contrast, a New Hampshire study^31^ found radiotherapy‐related BCC risk was higher in those without a sunburn history, suggesting radiation effects may be greater on less UV‐exposed skin, similar to findings from the Japanese A‐bomb study. Overall, however, differences in age at exposure, dose levels, and measurement methods limit direct comparisons across studies in Table 4.

## 5 CONCLUSIONS

This study provides evidence of increased BCC risk associated with increasing cumulative dose among UK radiation workers, but this association was substantially reduced after adjusting for anatomical site, indicating significant confounding by UVR. However, elevated BCC risk persisted among workers employed before 1960 or those monitored for internal exposure after adjustment for anatomical site, suggesting a residual radiation effect in subgroups with higher cumulative doses. Site‐specific analyses showed the strongest and only statistically significant association for BCC on the upper limbs. Non‐significantly raised risks were also observed for the scalp/neck and trunk, indicating modest site‐specific effects, possibly independent of UVR. In contrast, SCC showed only a weak and marginally significant association, largely driven by a small number of cases at high dose (above 800 mSv). Once these were excluded or site‐adjusted, the association disappeared, with no clear evidence of excess risk in external or internal exposure subgroups. Overall, this study reinforces that BCC, rather than SCC, is the NMSC subtype most consistently associated with occupational radiation exposure. These findings highlight the importance of adjusting for UVR exposure while also revealing a modest, residual radiation BCC risk in key subgroups, especially in earlier cohorts or those with prolonged exposure. Continued monitoring of radiation‐exposed populations and careful consideration of UVR confounding remain essential for accurate risk assessment and protection strategies. Overall, there was no strong evidence of an interaction between UVR and ionising radiation exposure in the development of NMSC. While UVR may act as a promoter in the development of skin cancer at moderate to high radiation doses —especially among individuals who are more sensitive to UVR and/or ionising radiation—there is currently no conclusive evidence that UVR modifies radiation‐related risk at low doses.

A major strength of our study in comparison with the other studies is the large cohort of workers chronically exposed to external radiation at low to moderate doses. The pathological verification of the NMSC cases enabled subgroup analyses by histological type and their anatomical sites, adding a substantial contribution to this field.

## AUTHOR CONTRIBUTIONS

**Nezahat Hunter:** Writing – original draft; formal analysis; investigation; writing – review and editing; methodology; software. **Richard Haylock:** Supervision; writing – review and editing; validation; investigation.

## FUNDING INFORMATION

The authors received no specific funding for this work.

## CONFLICT OF INTEREST STATEMENT

The authors declare no conflict of interest.

## ETHICS STATEMENT

The study has been subject to favorable REC review (South Central—Oxford C REC, ref.: 21/SC/0063). The study privacy notice can be found at: www.gov.uk/government/publications/radiation‐workers‐and‐their‐health‐national‐study/privacy‐notice‐for‐national‐registry‐for‐radiation‐workers‐nrrw.

## Supporting information


**Data S1.** Supporting Information.

## Data Availability

Access to data in the NRRW is via application to the NRRW Data Governance Group: https://www.gov.uk/government/groups/national‐registry‐for‐radiation‐workers‐governance‐group. Further information is available from the corresponding authors upon request.
