# Supplementary material for: The risk of basal and squamous cell carcinomas of the skin cancer incidence and external radiation in the updated National Registry for radiation workers cohort in the UK
Source: Int J Cancer. 2025 Sep 1;158(3):574–86. doi: 10.1002/ijc.70096 (PMC12670341; doi:10.1002/ijc.70096)

Supplementary materials

The risk of basal and squamous cell carcinomas of the skin cancer incidence and external radiation in the updated National Registry for Radiation Workers cohort in the UK

Nezahat Hunter and Richard Haylock

Table of contents:

Supplementary Table S1

Supplementary Table S2

Supplementary Table S3

Supplementary Figure S1

Supplementary Figure S2

Supplementary Table S1. Crude skin cancer incidence rates by histology according to cumulative dose (mSv) and the anatomical location.

	BCC							
	**Whole data**		**10 mSv**		**10-399 mSv**		**400+ mSv**	
	Total cases (%)	Rate *	Cases	Rate*	Cases	Rate*	Cases	Rate*
Lip	257 (6.0)	7.2	146	6.0	106	9.5	5	22.8
Face (eyelid and other face)	549 (12.8)	15.4	302	12.5	233	20.8	14	63.8
Ear	1658 (38.7)	46.6	895	37.1	731	65.2	32	145.7
Scalp & neck	509 (12.0)	14.3	290	12.0	215	19.2	4	18.2
Trunk	215 (5.0)	6.4	139	5.8	69	6.2	7	31.9
Upper limbs	135 (3.1)	3.4	74	3.1	60	5.4	1	4.6
Lower limbs	54 (1.3)	1.5	39	1.6	13	1.2	2	9.1
Overlapping lesion	873 (20.4)	24.5	562	23.3	301	26.8	10	45.5
NOS	38 (0.1)	1.1	26	1.1	10	0.9	2	9.1
**TOTAL**	4288	120.6	2473	102.5	1738	155.0	77	350.6
Person years	3,556,834		2,413,454		1,121,421		21,959	

*: Rate per 10^5 persons per year

Supplementary Table S2. Crude skin cancer incidence rates by histology according to cumulative dose (mSv) and the anatomical location.

	SCC							
	**Whole data**		**10 mSv**		**10-399 mSv**		**400+ mSv**	
	Total cases (%)	Rate*	Cases	Rate*	Cases	Rate*	Case s	Rate*
Lip	23 (2.8)	0.6	21	0.9	2	0.2	0	0
Face (eyelid and other face)	292 (35.7)	8.2	153	6.3	133	11.9	6	27.3
Ear	189 (23.1)	5.3	103	4.3	79	7.0	7	31.9
Scalp & neck	68 (8.3)	1.9	39	1.6	28	2.5	1	4.6
Trunk	111 (13.6)	3.1	57	2.4	53	4.7	1	4.5
Upper limbs	48 (5.9)	1.3	34	1.4	14	1.2	0	0
Lower limbs	6 (0.8)	0.2	4	0.2	2	0.2	0	0
Overlapping lesion	59 (7.3)	1.7	32	1.3	25	2.2	2	9.1
NOS	22 (2.7)	0.6	10	4.1	12	1.1	0	0
**TOTAL**	818	2.3	453	18.8	348	31.0	17	77.4
Person years	3,556,834		2,413,454		1,121,421		21,959	

*: Rate per 10^5 persons per year

Supplementary Table S3. The anatomical distribution of BCCs and SCCs in relation to external doses lagged 10-year.

Location	BCC (external workers sub-cohort)		SCC		
	Cases (female)	ERR/Sv (95% CI) a	Cases (female)	ERR/Sv (95% CI) a	ERR/Sv (95% CI) b
Lip	182 (15)	-0.98 (-2.36; 2.03)	23 (0)	5.55 (-9.1; 60.6)	-0.22 (-0.62; 1.20)
Face (eyelid and other face)	389 (20)	-0.46 (-1.88; 1.80)	292 (1)	0.22 (-0.65; 1.91)	0.05 (-0.07; >0.17)
Ear	1,177 (87)	-1.09 (-1.97; 0.02)	189 (6)	1.17 (-0.37; 3.79)	0.06 (-0.08; >0.15)
Scalp & neck	379 (17)	-0.55 (-2.64; 2.77)	68 (4)	1.34 (-1.41; 7.89)	0.32 (<0.03; >0.56)
Trunk	167 (5)	0.21 (-1.55; 4.48)	111 (4)	-0.92 (-1.23; 0.37)	0.06 (-0.11; >0.25)
Upper limbs	97 (17)	2.82 (-3.84; 15.4)	48 (10)	-1.90 (<-1.96; >-3.68)	-0.41 (-0.91; >-0.10)
Lower limbs	40 (0)	-7.11 (-11.4; 17.9)	6 (0)	57.6 (-113; 7246)	-0.37 (-1.21; >0.33)
Overlapping lesion	672 (45)	-0.57 (-1.20; 0.74)	59 (3)	0.42 (-1.43; 4.74)	0.20 (-0.06; >0.54)
NOS	27 (3)	10.9 (-12.4; 344)	22 (1)	-0.93 (<-33; >82.5)	0.59 (-0.71; 2.42)
Test for heterogeneity*					P>0.5*
Total	3,130 (209)	0.26 (-0.48; 1.18)	818 (29)	0.87 (0.03; 2.05)	0.37 (-0.27; 1.30)

a: Risk estimates based on restricted to those workers with specific anatomical location; b: Adjusted for anatomical location; *: Test of heterogeneity of the ERR/Sv across categories (not significant P>0.05)

Supplementary Figure S1. Anatomical sites distribution of BCC and SCC by cumulative external dose.

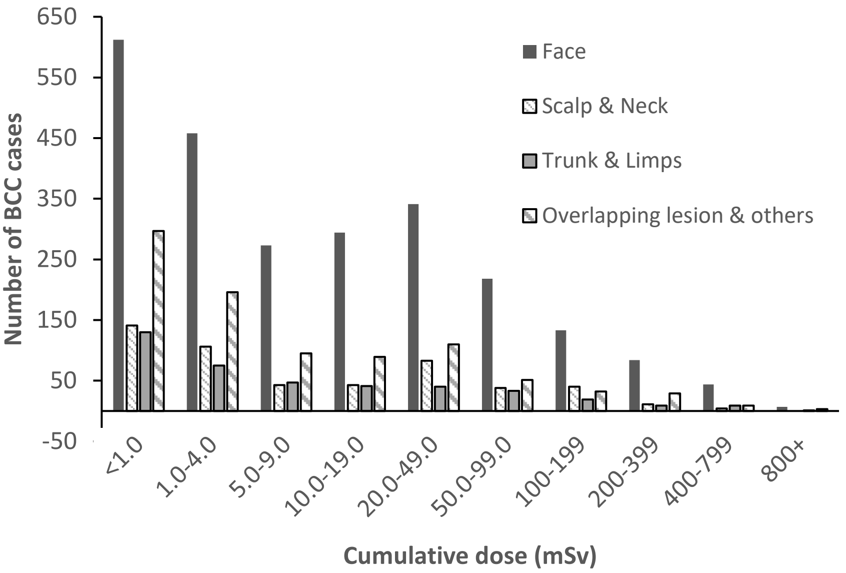

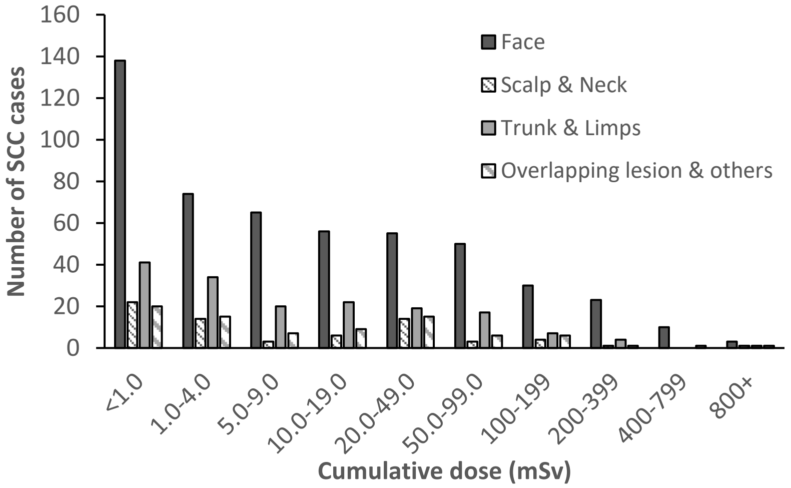

Supplementary Figure S2. The dose-response relationship in the low-dose range (0-100 mSv) for BCC or SCC, in the entire cohort and for BCC in the sub-groups: those also monitored internal exposure (Internal) and external exposure only (External)

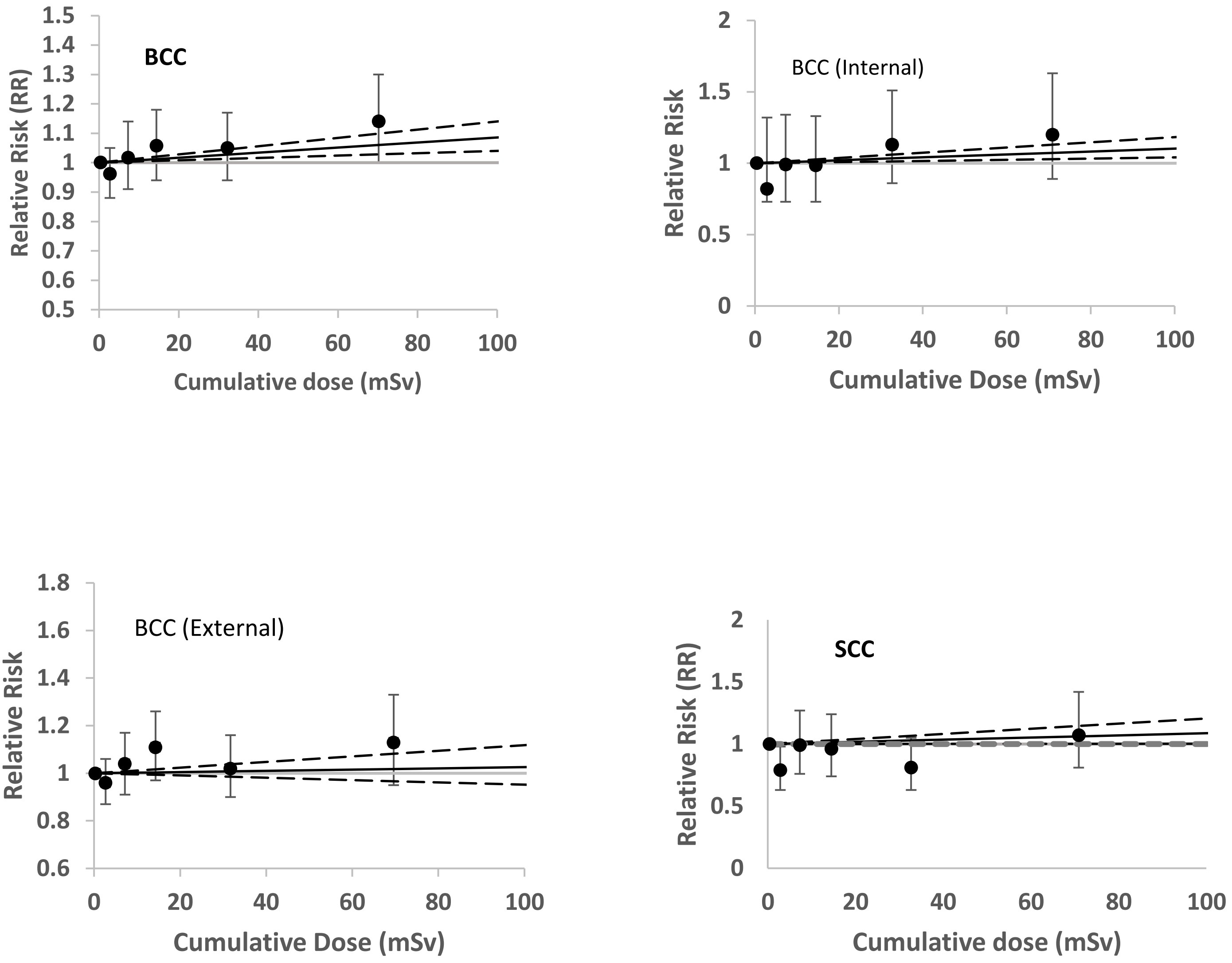

Supplement: Supplementary file 1 — Data S1. Supporting Information. [file IJC-158-574-s001.pdf]
